# Agent-based model provides insight into the mechanisms behind failed regeneration following volumetric muscle loss injury

**DOI:** 10.1371/journal.pcbi.1008937

**Published:** 2021-05-10

**Authors:** Amanda M. Westman, Shayn M. Peirce, George J. Christ, Silvia S. Blemker

**Affiliations:** 1 Biomedical Engineering, University of Virginia, Charlottesville, Virginia, United States of America; 2 Ophthalmology, University of Virginia, Charlottesville, Virginia, United States of America; 3 Orthopaedic Surgery, University of Virginia, Charlottesville, Virginia, United States of America; 4 Mechanical and Aerospace Engineering, University of Virginia, Charlottesville, Virginia, United States of America; Universiteit Leiden, NETHERLANDS

## Abstract

Skeletal muscle possesses a remarkable capacity for repair and regeneration following a variety of injuries. When successful, this highly orchestrated regenerative process requires the contribution of several muscle resident cell populations including satellite stem cells (SSCs), fibroblasts, macrophages and vascular cells. However, volumetric muscle loss injuries (VML) involve simultaneous destruction of multiple tissue components (e.g., as a result of battlefield injuries or vehicular accidents) and are so extensive that they exceed the intrinsic capability for scarless wound healing and result in permanent cosmetic and functional deficits. In this scenario, the regenerative process fails and is dominated by an unproductive inflammatory response and accompanying fibrosis. The failure of current regenerative therapeutics to completely restore functional muscle tissue is not surprising considering the incomplete understanding of the cellular mechanisms that drive the regeneration response in the setting of VML injury. To begin to address this profound knowledge gap, we developed an agent-based model to predict the tissue remodeling response following surgical creation of a VML injury. Once the model was able to recapitulate key aspects of the tissue remodeling response in the absence of repair, we validated the model by simulating the tissue remodeling response to VML injury following implantation of either a decellularized extracellular matrix scaffold or a minced muscle graft. The model suggested that the SSC microenvironment and absence of pro-differentiation SSC signals were the most important aspects of failed muscle regeneration in VML injuries. The major implication of this work is that agent-based models may provide a much-needed predictive tool to optimize the design of new therapies, and thereby, accelerate the clinical translation of regenerative therapeutics for VML injuries.

## Author summary

For common muscle injuries, such as lacerations or strains, skeletal muscle has the ability to repair itself through a series of highly orchestrated cellular processes. However, in the case of volumetric muscle loss (VML) injuries, a large amount of muscle is removed and the muscle’s intrinsic regenerative process fails resulting in the injury filling with fibrotic tissue. Currently there are no therapies that adequately repair muscle tissue for VML injuries, and a contributing factor is that the cellular mechanisms driving the response to these injuries are poorly understood. To aid in addressing this knowledge gap, we have developed an agent-based model to capture the cellular remodeling processes following the creation of a VML injury. We have demonstrated that our model is capable of predicting the key aspects of tissue remodeling following VML injury. Moving forward, our model can be used as a predictive tool to assess the ability of new therapies to repair VML injuries and thereby accelerate the development of improved treatments to the clinic.

This is a *PLOS Computational Biology* Methods paper.

## Introduction

In response to common injuries, such as lacerations or strains, skeletal muscle repair occurs through a well described process governed by temporally-regulated, highly orchestrated, multicellular interactions [1,2]. However, in the setting of volumetric muscle loss (VML) injuries, which are characterized by the simultaneous loss of multiple tissue compartments (i.e., muscle, vessel, nerve and extracellular matrix (ECM)), the intrinsic regenerative process fails, resulting in permanent cosmetic and functional deficits. VML typically results from trauma, such as battlefield injuries to wounded warriors, or civilian vehicular accidents and there are no current treatment options for complete restoration of form and function. In this scenario, the lack of insight into the mechanisms responsible for the failure of functional regeneration, in the context of VML injury, represents a major barrier to development of novel therapeutics for improved functional outcomes.

**Fig 1:**
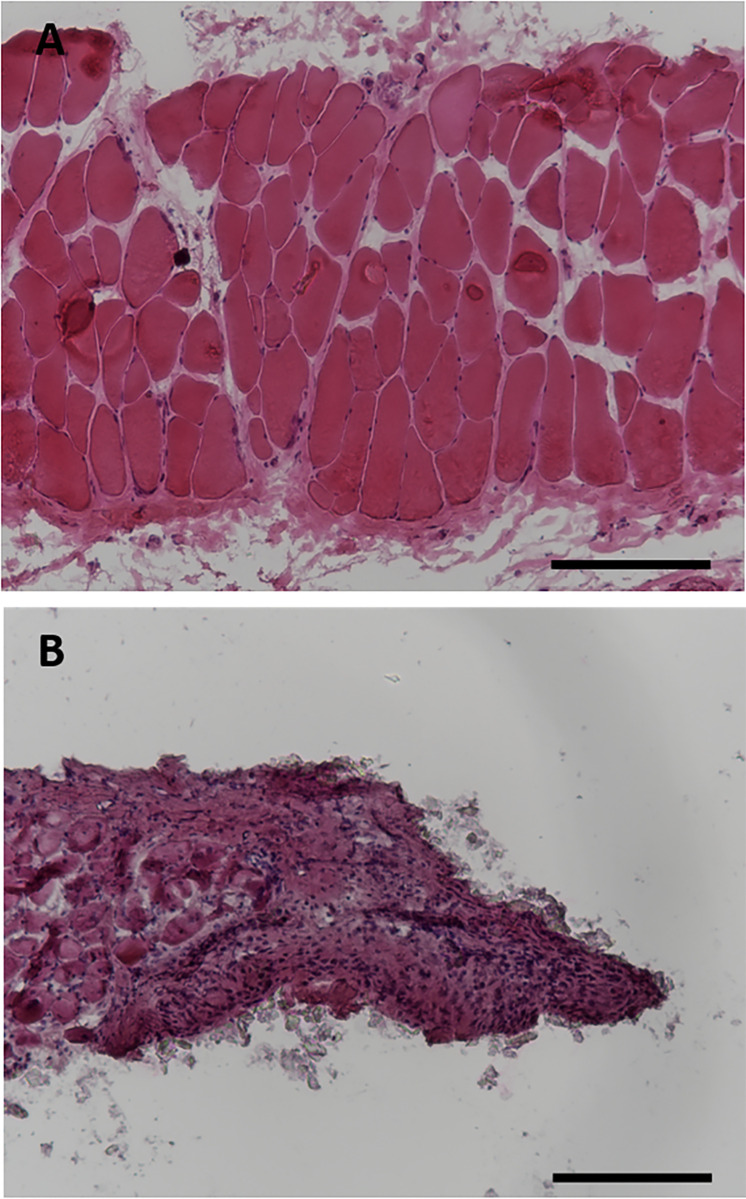
Histology of hematoxylin and eosin-stained rat latissimus dorsi skeletal muscle shows the cross-section of healthy muscle (A) and the cross-section 7 days after VML injury (B). The lack of muscle fibers on the right half of the image marks the defect caused by the VML injury. Scale bars = 200 μm.

Successful muscle tissue repair in response to lacerations, strain, or toxin induced injuries generally occurs within 28 days and involves 3 well documented phases [3–5]. An initial inflammatory response coordinated by an infiltration of neutrophils and pro-inflammatory (M1) macrophages to clear debris. The second phase, repair, follows with fibroblast and satellite stem cell (SSC) activation and proliferation. Within 7 days post injury, macrophages switch to an anti-inflammatory (M2) phenotype and fibroblasts and SSCs numbers peak. After 14 days, the remodeling (third) phase begins and fibroblasts undergo apoptosis and SSCs differentiate and fuse to repair existing myofibers. In contrast, the repair process of skeletal muscle after VML injury is dominated by an inflammatory and fibrotic response [6–8], resulting in permanent loss of muscle volume, replacement of muscle with scar tissue, and resulting functional impairments. Following VML injuries, pro-inflammatory pathways remain upregulated for four weeks, as opposed to one week in successful muscle repair [9]. SSCs fail to differentiate, and fibroblasts and myofibroblasts do not undergo apoptosis like in other muscle injuries but instead fill the defect space with fibrotic tissue, which includes densely packed collagen (Fig 1) [3,9–11].

In short, VML injuries require therapeutic intervention for complete functional regeneration. Both preclinical studies [11–13], as well as limited initial clinical trials [14,15], clearly indicate there is a need for improved therapeutic design. Strategies evaluated thus far have included implantation of decellularized ECM, minced muscle grafts (MMGs), as well as a variety of natural and synthetic biomaterials seeded with muscle progenitor cells and/or a combination of growth factors to help promote SSC proliferation and differentiation [11–13,16–19]. The results have been variable, but inclusion of cells with decellularized ECM at the time of implantation has generally shown greater promise in functional restoration of muscle tissue after VML injury than implantation of decellularized ECM alone [12,13,19,20]. Despite rapidly increasing preclinical activity, important questions remain about which early cellular processes are required to more effectively drive the VML injury response. For example, will modulating the early inflammatory response or mitigating fibrotic pathways in VML injuries, or both, improve muscle regeneration and functional outcomes? A better understanding of these mechanisms is a prerequisite to the design of more efficacious regenerative therapeutics for VML repair [8,9,21–25].

Experimental identification and validation of the critical cellular mechanisms and microenvironmental conditions associated with the tissue healing response to VML injury is a resource-intensive and time-consuming process. The deployment of agent-based models (ABMs) in parallel with experiments provides a powerful way to synthesize and integrate data in order to systematically predict, in a more rational and cost-effective manner, how cellular mechanisms of interest impact tissue repair/remodeling and optimally affect regeneration [26]. ABMs simulate cellular behaviors and show the effects of these cellular behaviors on the physiological system as a whole. Agents represent individual cells within a tissue as well as environmental components, such as ECM, and the computational platform synthesizes the published work in the field, as agent behaviors are governed by literature-derived rules [27]. Our group has previously developed an ABM of muscle regeneration that was focused on the role of inflammatory cells following laceration injury [28]. We subsequently modified this model to study how different Duchenne muscular dystrophy mechanisms influence muscle regeneration [29]. In this study, we are expanding the use of ABMs to study the cellular responses following VML injury.

The goal of this work was to develop an ABM of muscle regeneration in the setting of VML injury that focused on the dynamics of fibroblasts and SSCs in order to better understand the critical cellular mechanisms responsible for regeneration. We first used the ABM to predict the tissue healing response in an unrepaired VML injury, and then we tuned the model to replicate important cell population dynamics from published experimental studies. The ABM was validated by simulating VML injury and tissue healing/repair following implantation of either an acellular biomaterial (decellularized ECM) or another therapeutic that included a cellular component (MMG). In both cases the model simulations were compared to published experimental data. Lastly, in order to identify new strategies for muscle regeneration following VML, we evaluated the impact of perturbation of parameters and combinations of parameters of interest, all of which had defined cell behaviors known to be important in muscle regeneration.

## Methods

### ABM design

**Fig 2:**
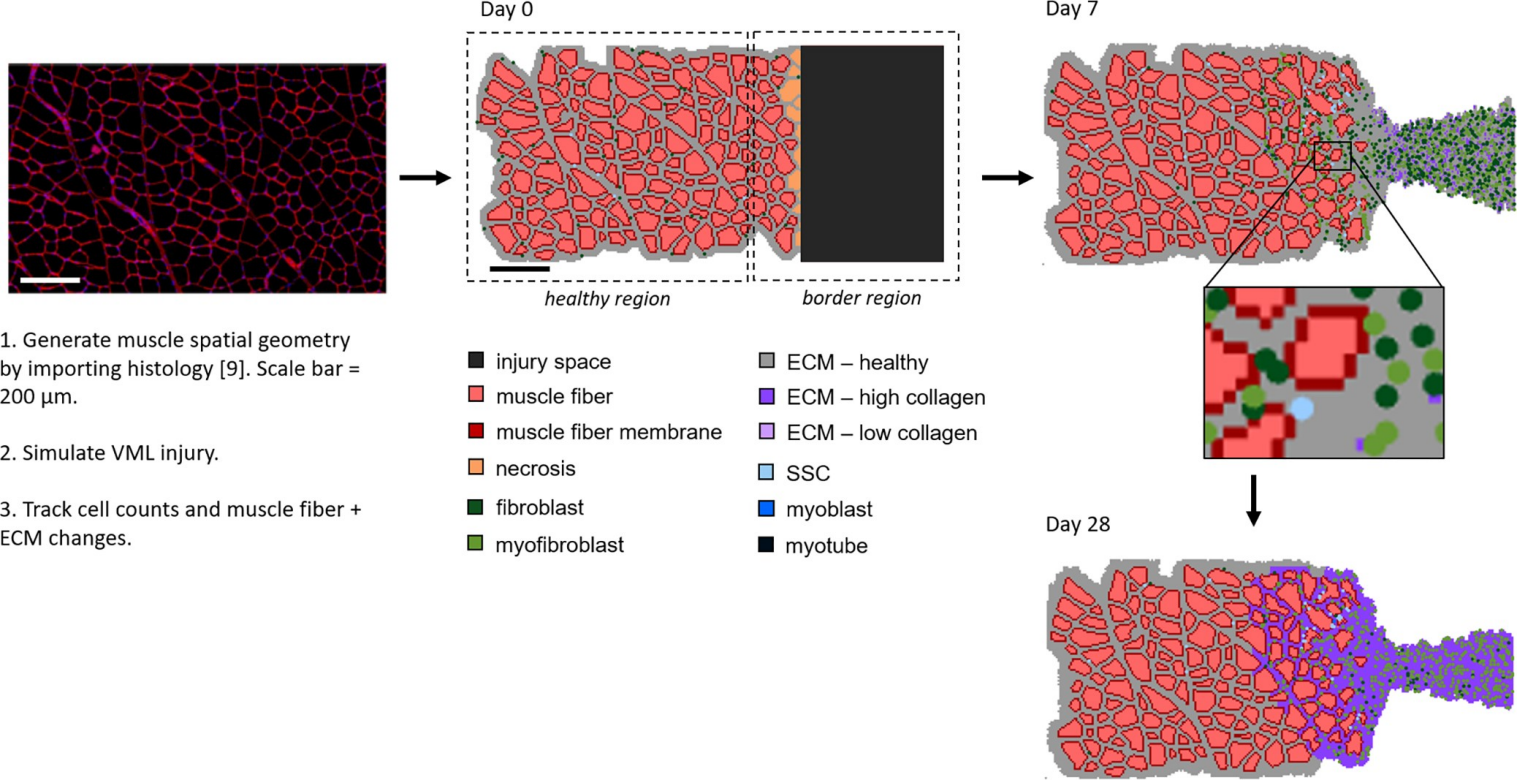
ABM simulates muscle regeneration for 28 days following VML injury. The spatial geometry of the ABM was defined by importing a histological image [9]. Then a VML injury was simulated by removing 12 fibers and creating an injury space, and replacing severed fibers at the edge of the injury and native muscle with necrotic elements. The ABM consisted of two regions–the healthy region of muscle fibers near the injury that were not affected by the defect and the border region consisted of fibers near the injury and the injury space. Regeneration was followed over time by tracking cell counts, muscle fiber counts, and collagen density in each region.

We created an ABM of skeletal muscle regeneration following VML injury (Fig 2). To develop our model, we used over 100 published experimental studies to define 80 rules that govern the behaviors of fibroblasts, SSCs, inflammatory cells, and skeletal muscle. Our approach was inspired by previously published ABMs of biologic systems [26,28–33]. The ABM represented a two-dimensional cross-section of a rat skeletal muscle consisting of 164 muscle fibers. The agents that occupied space in the model included muscle fibers, ECM, necrotic muscle tissue, fibroblasts, myofibroblasts, quiescent and activated SSCs, myoblasts, myocytes, and myotubes. Model components whose spatial location was not tracked included eleven growth factors and three types of inflammatory cells: neutrophils, pro-inflammatory (M1) macrophages, anti-inflammatory (M2) macrophages. We built the ABM in Repast, a java-based modeling platform (Argonne National Laboratory, Lemont, IL) and the ABM’s code is available for download (https://simtk.org/projects/abm-vml). The code download package includes all commented source code, visualization code, and necessary support files to run the VML injury ABM in Repast.

Overall, the ABM was a two-dimensional grid of over 30,000 pixels including the muscle fibers and injury area (1.59 x 0.74 mm^2^). The muscle cross-section geometries in the ABM were generated by importing a micrograph of a histological section of muscle (1.3 x 0.74 mm^2^, 10 μm thick) with 188 fibers [9,34]. The image was first processed in MATLAB (Mathworks, Natick, MA) to generate a file consisting of each pixel labeled as fiber, fiber border, or ECM. This labeled file was then imported into the ABM grid. The VML injury was created at the start of the simulation by removing 12 imported muscle fibers on the right edge of image and adding pixels to create the injury defect. Fibers that were severed by the creation of the injury were automatically replaced with necrotic agents. After injury and initial necrosis, the muscle area was 1.1 x 0.74 mm^2^ (164 fibers) and the injury area was 0.49 x 0.74 mm^2^. The injury area is larger than the area of the cut fibers to ensure that the ratio of remaining muscle area to injury space was comparable with the experimental studies we used to tune our model (approximately 20% VML injury) [9,35].

In VML injuries, the spatial relationship between cells relative to the VML defect location affects their responses during the regeneration process; therefore, the ABM consisted of a healthy muscle region and a border region that was adjacent to the defect. The healthy region consisted of the muscle fibers near the VML injury that were not affected by the defect, approximately 130 μm from the injury space [36]. The border region consisted of the fibers within 130 μm of the injury and the injury space [11,17,36]. This ABM spatial representation and the baseline number of cells (e.g. fibroblast, SSC) were defined when the ABM was initialized.

**Fig 3:**
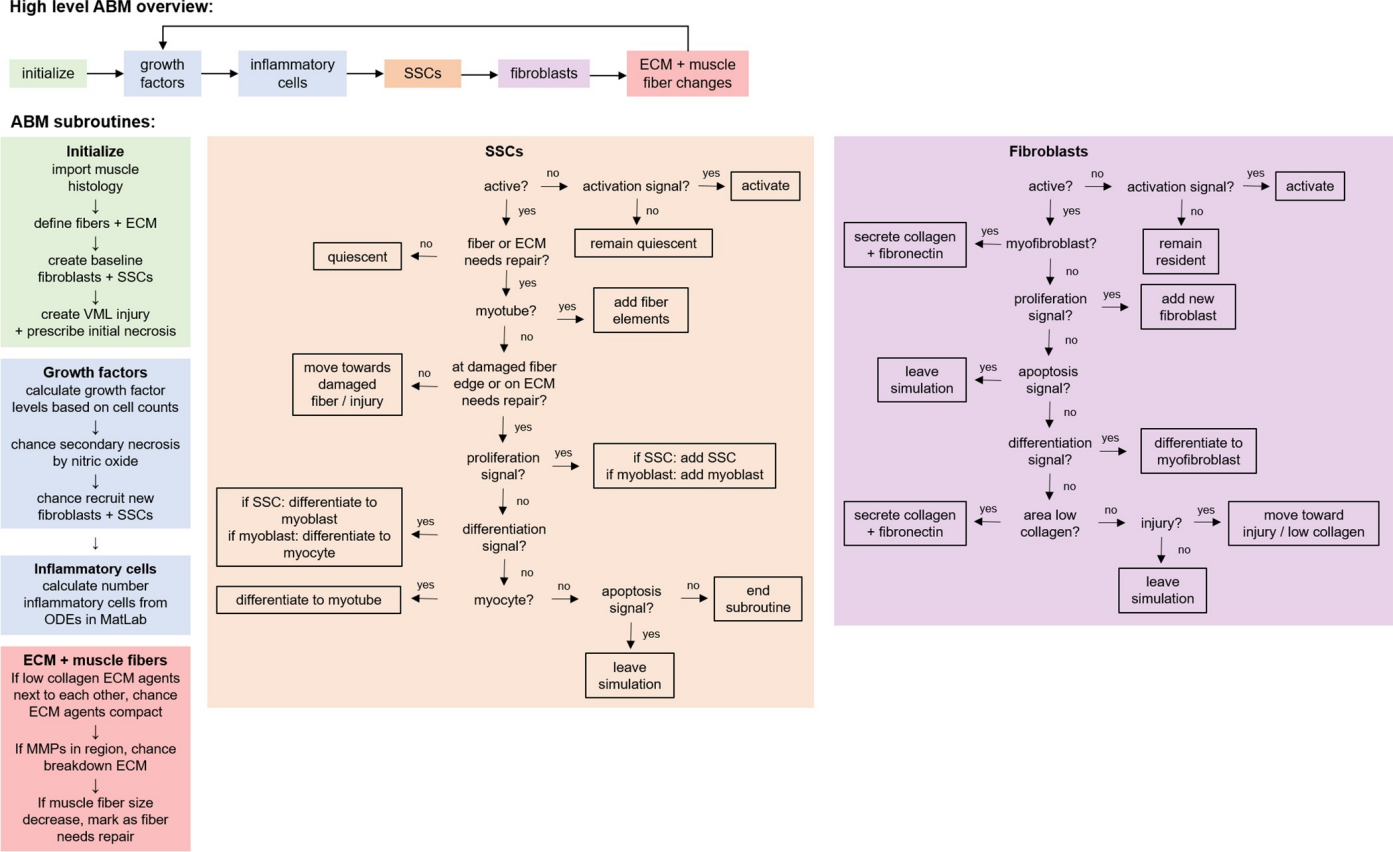
Flowchart depicts the ABM rules, logic flow, and agent actions. After initialization, the growth factors and inflammatory cells are calculated during each subsequent time step. Then SSCs, fibroblasts, fibers, and ECM follow a probability-based decision tree to guide their actions.

Simulations were run with a 1-hour time step for a simulated 28 days following injury to capture the timeframe of typical muscle repair (see S1 Video for animation) [3–5]. At each time step of the simulation, every cellular agent (e.g. fibroblast, SSC) determined its location in the simulation space and then its behaviors were determined by a probability-based decision tree (Fig 3). As an example of cellular behavior occurring at every time step, the following description describes the behavior of a SSC agent defined by its probability-based decision tree. A SSC agent first determined if it was active. If it was not active, the SSC agent had a chance of activating corresponding to the activation signal in its region. If it was already active and there was an injury in the simulation space, the SSC agent would move towards the damaged fiber or injury. If the active SSC agent was already at the injury location, then there was a probability it would proliferate based upon the strength of the SSC proliferation signal. There was also a probability the active SSC agent near the injury would differentiate corresponding to the strength of the SSC differentiation signal. If the SSC agent was already a fully differentiated myotube, then it would put down a new fiber element. The strength of the SSC apoptosis signal influenced the likelihood of the SSC agent undergoing apoptosis and leaving the simulation. For model components whose autonomous behavior was not modeled (inflammatory cells, growth factors), their counts in each region were tracked and contributed to autonomous agent behavior as described in the following sections. The collective actions of all the autonomous agents (fibroblasts, SSCs, ECM, muscle fibers) lead to emergent, system-level behaviors (fiber counts, cell population dynamics) that were output by the simulations. All simulations were repeated 10 times to capture the stochastic cell behaviors of the model. The key model outputs included the time-varying counts for each cell type in the model and the number of muscle fibers.

### Agent actions

The simulated behaviors of fibroblast and SSC agents included secretion of growth factors, quiescence, activation, recruitment, migration, proliferation, differentiation, and apoptosis. To simulate migration, the agents were programmed to move to a neighboring pixel by randomly selecting a neighbor within the limits of experimentally derived migration rates (Tables 1 and 2). Quiescent agents did not migrate or secrete growth factors until they were activated by growth factors. If an agent was recruited to the injury, then a new agent was added to the simulation at a random pixel location. An active agent could undergo migration, secretion of growth factors, proliferation, differentiation, and apoptosis. Agent proliferation was represented by adding an additional agent to the simulation next to the proliferating agent. For the simulations included in this work, there is no rule directly preventing cell overlap in the simulation. S1 Fig demonstrates that preventing cell-cell overlap does not significantly influence the model outputs. Agent differentiation was represented by changing the agent type to the differentiated state (rules that govern differentiation behaviors and states are defined in the fibroblast and SSC agents subsections). If an agent underwent apoptosis, it was removed from the simulation. Agent behavior was influenced by behavior signals that were defined by a function of factors that are known to promote or suppress each behavior (Eq 1). The cell behavior signal was then normalized by a factor accounting for the size of the ABM grid, and the probability of a cell behavior occurring was weighted by this behavior signal (Eq 2) [28,29]. For example, larger cell behavior signals increased the probability of a cell behavior. Each cell behavior probability had a maximum probability value to preserve the stochastic nature of cell behaviors. If there were very large cell behavior signals, than there was a limit to the probability of a behavior occurring. The maximum probability value for cell behaviors were tuned during parameterization of the no repair ABM.

cellbehaviorsignal=∑factorspromote‐∑factorssuppress (1)

$$\mathrm{cell}\;\mathrm{behavior}\;\mathrm{probability}=\frac{1}{\mathrm{normalization}\;\mathrm{factor}-\mathrm{cell}\;\mathrm{behavior}\;\mathrm{signal}}$$ (2)

**Table 1:** Fibroblast agent behaviors were defined by literature derived rules.

*Fibroblast Agent Behavior*	*References*
Initial count: 1 per every 3 fibers	[37–39]
Recruitment signal: eosinophil recruited IL-4	[8,40]
Migrate toward injury/low collagen at rate of ~44 μm/h	[8,37,41]
Proliferation signal: myostatin from damaged ECM	[42–44]
Differentiation signal: TGF-β	[7–9,45]
Apoptosis signal: TNF-α If sustained high levels of TGF-β, then blocked apoptosis	[9,46]
Generalized decrease (removal) of fibroblast following injury, e.g. adipogenic differentiation	[46–48]
Secretions: TGF-β, IGF, IL-6, FGF, MMPs	[48,89–94]
Secrete collagen + fibronectin to rebuild ECM and fill injury space	[66,94]
Myofibroblast secrete more collagen than fibroblasts and TGF-β, MMPs	[7,9,13,45,95]
If > 200 μm from healthy muscle, then fibroblasts and myofibroblasts decrease collagen secretion + migration speed	[49–51]

**Table 2:** SSC agent behaviors were defined by literature derived rules.

*SSC Agent Behavior*	*References*
Initial count: 1 per every 23.5 fibers	[37,52]
Activation signal: HGF, released from ECM after injury	[53–56]
Recruitment signal: HGF + IGF + FGF + MMP– 2*TGF-β	[96–99]
Migrate to injury site at ~50 μm/h	[11,17,57,58]
Migrate if MMP degrade ECM	[98,100]
Microenvironmental cues for proliferation + differentiation: on fiber edge, on ECM with stiffness similar to healthy muscle	[69–71,101]
Proliferation signal: 3*IGF + 3*FGF + HGF + TNF-α + IL-6 + IFNγ–IL-10–4*TGF-β	[7,55,56,102–104]
Proliferation attenuated by sustained upregulation of inflammatory response	[37]
Differentiation signal: 4*IL-10–2*FGF– 2*IGF– 2*HGF–IFNγ–TNFα	[97,103,105,106]
10% of SSCs do not express Myf5 and will not differentiate	[62–64]
Activated SSCs differentiate to myoblasts, myoblasts to myocytes, and myocytes to myotubes	[54,107,108]
Myotubes put down muscle protein to repair existing fiber or put down new fiber	[59–61]
Apoptosis signal: TGF-β	[109]
Secretions: IL-6, MMPs, IL-1	[110,111]

### Fibroblast agents

**Fig 4:**
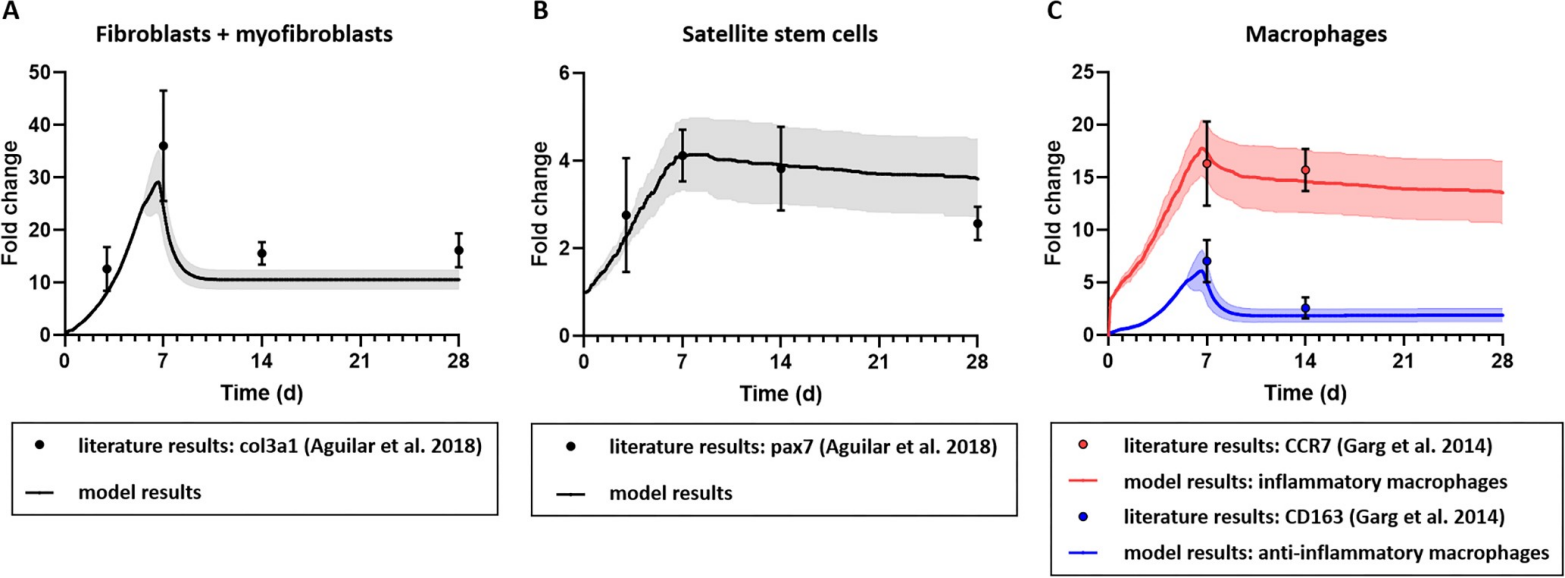
ABM of VML injury regeneration without repair parameterized to capture experimentally reported cell population behaviors. The model replicated an experimentally measured fold change in (A) the number of fibroblasts and myofibroblasts, (B) SSCs, and (C) M1 and M2 macrophages, within the model’s predicted 95% confidence interval. Model results were reported as mean ± 95% confidence interval. Aguilar et al. 2018 experimental data of fibroblasts and satellite cells reported as median ± standard deviation, and Garg et al. 2014 experimental data of macrophages reported as mean ± standard error mean [9,23].

During model initialization, the fibroblast agents were distributed randomly throughout the ECM at a density of 1 fibroblast per every 3 fibers for a 10 μm thick cross-section [37–39]. After VML injury, additional fibroblast agents were recruited at a rate that was proportional to the amount of IL-4 secreted by eosinophils [8,40]. Fibroblasts migrated to the injury site at a rate of 44 μm/h and had a preference to move to low collagen ECM agents [8,37,41]. The growth factor myostatin promoted proliferation of fibroblast agents [42–44], and high levels of TGF-β promoted an increased likelihood of fibroblast agents differentiating to myofibroblasts [7–9,45]. The likelihood of fibroblast apoptosis was elevated by the presence of TNF-α, whereas TGF-β blocked apoptosis [9,46]. There is experimental evidence that fibroblast counts decrease after injury, and studies have shown that this is partially a result of differentiation to adipocytes but the mechanism is not clear [46–48]. We incorporated a fibroblast removal rule to capture this behavior and the likelihood of fibroblast removal in the simulation was tuned to capture the experimental data of declining fibroblast counts after injury (ABM parameterization subsection, Fig 4A). Fibroblast and myofibroblast agents secreted growth factors and collagen following injury (Table 1). If the fibroblast or myofibroblast agent was greater than 200 μm from healthy muscle, then the agent became hypoxic and the collagen secretion and migration speed decreased [49–51]. Other effects of hypoxia, such as the release of apoptotic factors, were not included in the model for the mechanisms of fibroblast count decrease after injury are not clear, as mentioned above, and our goal was to focus on the growth factors secreted after injury.

### SSC agents

During initialization, the SSC agents were randomly located at a muscle fiber edge in a quiescent state at a density of 1 SSC per 23.5 muscle fibers for a 10 μm thick cross-section [37,52]. Following VML injury, SSC agents became activated by ECM damage and the presence of hepatocyte growth factor [53–56]. Recruitment of SSC agents to the injury site was based on a recruitment signal of growth factors (Table 2). Migration of activated SSCs toward the injury site occurred at a rate of 50 μm/h [11,17,57,58]. SSC agents proliferated based on the microenvironmental cues and growth factors outlined in Table 2. If there was sustained upregulation of the inflammatory response, then SSC proliferation was attenuated [37]. Terminal differentiation of SSC agents was based on a differentiation signal and microenvironmental cues (Table 2). To simulate regeneration, differentiated myotubes could add muscle fiber agents to the periphery of an injured fiber or deposit fiber agents to generate a new fiber depending on its location [59–61]. Approximately 10% of the SSC population was instructed not to undergo differentiation, and helped to restore the SSC agent pool [62–64].

### ECM and muscle fiber agents

At initialization, a single muscle fiber was represented by an average of 72 pixels. Muscle fiber agents were removed along the edge of the ABM grid to represent VML injury, and the fibers which were cut were replaced by necrotic agents. During the simulation, secondary necrosis spread based on nitric oxide levels secreted by inflammatory cells [2,65]. The rate of necrosis agent removal was dependent on the number of M1 macrophage agents [7,65]. Agents representing cleared necrosis were converted to a low-density collagen ECM agent. Fibroblast agents secreted collagen in low collagen areas and in the injury space [65,66]. If areas of very low collagen remained, then two neighboring ECM agents with low collagen had a probability of merging into a single agent. This simulated behavior reduced the thickness of the muscle cross-section near, and within, the injury site, which has been observed experimentally [67]. Areas of high-density collagen ECM agents corresponded to fibrotic tissue [11,68]. When differentiated myotubes were fused to a fiber edge that had been damaged through necrosis, muscle fiber agents were added at the periphery of the fiber to increase the muscle fiber size. If the myotubes were on an ECM agent with healthy levels of collagen (defined as a model specific range), then there was a chance of putting down a fiber agent to generate new muscle fibers. For ECM agents, a healthy level of collagen was defined to be a range of model-specific values that corresponded with temporal observations of low fibrosis levels in experimental histology [11,18,67]. Given the parameters in our model, we assumed that ECM levels of collagen correlated to muscle stiffness and thus this microenvironmental rule captured the SSCs dependency on healthy muscle stiffness to proliferate and differentiate [69–71].

### Growth factors

The 11 growth factors in the model (Tables 1 and 2) were varied from baseline levels (before injury) to levels following VML injury. Growth factors were tracked in each region of the model, healthy versus border regions, and added at each time step based on the defined secretions for each cell type in their respective region. The amount of growth factors within each region were tracked over time. We have assumed that the growth factors are evenly distributed in each region, they do not diffuse or move within the region, and they decay over time at a fixed half-life of 5 hours that represents the growth factors diffusing out of the tissue [3,72–75].

### Inflammatory cell ordinary differential equations

The inflammatory cell dynamics were defined based on previous work by Martin et al. and Virgilio et al. [28,29]. Our goal was to incorporate the dynamic behaviors of inflammatory cells but also reduce the computational cost of the ABM. The inflammatory cells were represented as a system of three coupled ordinary differential equations (ODEs) for neutrophils (N), M1, and M2 macrophages. The ODEs were defined by 20 parameters that represent the effect of one cell type on another cell. A genetic algorithm (GA) was used to parameterize the ODEs by minimizing the difference between ABM macrophage populations predicted by the model and experimentally measured macrophage dynamics after VML injury [17,23]. Each generation had 500 individuals with 20 variables. The GA objective function [Eq 1] was a sum of squared differences between simulation results (subscript ABM) and experimental data (subscript EXP) (Eq 3).

$$\mathrm{Objective}\;\mathrm{Function}=\sum_{t=1}^{672}{\left(N_{\mathrm{ABM}}-N_{\mathrm{EXP}}\right)}^{2}+\sum_{t=1}^{672}{\left({M1}_{\mathrm{ABM}}-{M1}_{\mathrm{EXP}}\right)}^{2}+\sum_{t=1}^{672}{\left({M2}_{\mathrm{ABM}}-{M2}_{\mathrm{EXP}}\right)}^{2}$$ (3)

To compare our hourly model predictions with the discrete experimental observations, we fit the experimental macrophage population dynamics with a fourth order polynomial equation (MATLAB). Each comparison was weighted by the variance of the experimental data. Variance for fitted time points was determined using a linear interpolation between experimental time points. We used a MATLAB GA solver (GA) where GA individuals that had the lowest objective function scores were used as parents for the next generation of individuals (20% offspring, 80% new random individuals). The GA was designed to stop if the objective function failed to decrease 1x10^-9^ for 50 consecutive generations, with a limit of 1000 generations.

Initially, we found values for the unknown ODE parameters using the genetic algorithm, experimental data, and ideal ABM output curves for percent necrosis, fibroblasts, and SSCs. Given the interdependency of inflammatory cell counts and other cell counts, ideal ABM output curves were used to eliminate one source of variability. We ran multiple GA iterations to fine tune the parameter solutions (S1 Table). The inflammatory ODEs were then run in conjunction with the ABM, and GA-tuned ODE parameter values did not accurately capture experimental inflammatory cell counts due to the stochastic nature of fibroblasts and SSCs in the ABM. We then manually tuned the inflammatory cell parameters until the ODE curves were within the standard deviation of experimental counts (S1 Table). The iterative process used to arrive at the ODE parameter values and the ability of the ODEs to accurately predict inflammatory counts with different ABM perturbations provides confidence that we have found a unique solution to the ODEs.

Within the ABM simulation framework, the inflammatory cell ODEs were solved for each region (healthy and border) by calling the MATLAB engine and using a MATLAB non-stiff differential equation solver, ode45. The regional breakdown of inflammatory cells was approximated to be 60% in the border region and 40% in the healthy region based on quantified data from a previously published study [17]. To couple the inflammatory cell ODEs with the behaviors of the other spatial cell agents, the inflammatory cell agents had defined rules based on cell counts at the beginning of each time step. The inflammatory cells secreted growth factors and removed necrosis agents, and their ODEs were dependent on the spatial cell agent counts at each time-step. Inflammatory cell ODEs (Eq 4–6) include the following, where %_necrosis_ is the current ratio of muscle that is necrotic, Fb is the current number of fibroblast agents, and SSC is the current number of SSC agents (constants are listed in Table 3):

$$\frac{\mathrm{dN}}{\mathrm{dt}}=A_{\%}*{\%}_{\mathrm{necrosis}}-A_{Fb}*\mathrm{Fb}-A_{N}*N-A_{M1}*M1-A_{M2}*M2-A_{SSC}*\mathrm{SSC}+A_{N\%}*N*{\%}_{\mathrm{necrosis}}$$ (4)

$$\frac{\mathrm{dM1}}{\mathrm{dt}}=B_{\%}*{\%}_{\mathrm{necrosis}}+B_{Fb}*\mathrm{Fb}+B_{N}*N-B_{M1}*M1-B_{M2}*M2+B_{SSC}*\mathrm{SSC}+B_{M1\%}*M1*{\%}_{\mathrm{necrosis}}$$ (5)

$$\frac{\mathrm{dM}2}{\mathrm{dt}}=C_{\%}*{\%}_{\mathrm{necrosis}}+C_{Fb}*\mathrm{Fb}+C_{N}*N-C_{M1}*M1-C_{M2}*M2+C_{SSC}*\mathrm{SSC}$$ (6)

**Table 3:** Constants for inflammatory cell ODEs.

*Constants*	*Value*
*Neutrophils*	
A_%_	52.08
A_Fb_	0.0021
A_N_	0.28
A_M1_	0.0023
A_M2_	0.012
A_SSC_	0.0022
A_N%_	0.065
*M1 Macrophages*	
B_%_	2.24
B_Fb_	0.21
B_N_	0.05
B_M1_	1.03
B_M2_	0.42
B_SSC_	23.31
B_M1%_	0.03
*M2 Macrophages*	
C_%_	126.73
C_Fb_	1.91
C_N_	0.07
C_M1_	0.74
C_M2_	9.07
C_SSC_	7.96

### ABM parameterization

To parameterize the baseline (no repair) model of VML injury, we ran simulations and systematically adjusted the unknown model parameters manually (Table 4) until the model predictions (95% confidence intervals) were consistent with published experimental data, which included fibroblast (marked by col3a1) and SSC (marked by Pax7) fold changes [9] and inflammatory cell fold changes (inflammatory macrophages marked by CCR7, anti-inflammatory macrophages marked by CD163) [23]. Tcf4+ is a fibroblast marker that has been reported to be specific to skeletal muscle [38]; however, there were no VML studies that have quantified expression of this marker. Thus, we fit our fibroblast and myofibroblast fold changes to the observed changes in gene expression of col3a1 (collagen 3) following VML injury [9] and it has been used as a marker of fibroblasts in other tissues [76–78].

**Table 4:** Unknown model probability parameters (x) were tuned to recapitulate published experimental results. P is probability and P_max_ is maximum probability.

*Probability Parameter*	*Equation*	*Range Tested*	*Value*
P_max_ of fibroblast recruitment, x_1_	If P of fibroblast recruitment > x_1_, recruitment probability = x_1_	0.0200–0.2000	0.0333
P_max_ of fibroblast proliferation, x_2_	If P of fibroblast proliferation > x_2_, proliferation P = x_2_	0.0200–0.5000	0.0286
P_max_ of fibroblast differentiation, x_3_	If P of fibroblast differentiation > x_3_, differentiation P = x_3_	0.0100–0.2000	0.0125
P_max_ of fibroblast apoptosis, x_4_	If P of fibroblast apoptosis > x_4_, differentiation P = x_4_	0.0100–0.2000	0.0125
P_max_ of fibroblast removal, x_5_	If P of fibroblast removal > x_5_, removal P = x_5_	0.0200–0.2000	0.1000
P_max_ of SSC recruitment, x_6_	If P of SSC recruitment > x_6_, recruitment P = x_6_	0.0008–0.0400	0.0008
P_max_ of SSC proliferation, x_7_	If P of SSC proliferation > x_7_, proliferation P = x_7_	0.0100–0.5000	0.0400
P_max_ of SSC differentiation, x_8_	If P of SSC differentiation > x_8_, differentiation P = x_8_	0.0100–0.5000	0.0666
P_max_ of SSC apoptosis, x_9_	If P of SSC apoptosis > x_9_, apoptosis P = x_9_	0.0020–0.5000	0.0020
P of SSC creating new fiber on ECM with healthy levels of collagen, x_10_		0.0100–0.0400	0.0200

Model predicted cell counts were normalized by the number of cells at initialization to calculate the fold change and allow for direct comparison with values reported in experimental studies. When comparing the ABM predicted cell counts with published experiments, we focused on 7 to 14 days post injury because this is when the counts peak according to the literature [9,23].

### Validation of ABM

Once the model parameters were tuned, we ran simulations to verify the model predictions and validate the ABM using data from the literature that was distinct from the data used for model calibration. We first simulated the administration of the anti-fibrotic agent, Losartan, to baseline (no repair) VML injuries [23]. Losartan was modeled by reducing TGF-β secretion from myofibroblasts and macrophages by 60% [79,80]. We assumed TGF-β production was impaired by 60% for Losartan inhibits a receptor in the angiotensin pathway which thereby blocks TGF- β activation and signaling; however, there are other pathways involved in TGF- β production and signaling thus we chose to reduce but not eliminate TGF- β production [23,79]. Additionally, an *in vivo* study quantified a 60% reduction of myostatin, a highly conserved TGF-β superfamily member, with Losartan treatment further confirming our assumption for TGF-β activity [80,81]. We then simulated two VML treatments that have been experimentally tested [9,11,13,35]: implantation of either a decellularized ECM or a MMG at the site of injury. We simulated decellularized ECM by filling the injury space with ECM agents at initialization [11,13]. MMG consists of autologous muscle minced into 1 mm^3^ pieces, and it was modeled by filling the injury space with 38 muscle fibers [9,13,35]. Twenty percent of the fiber agents were replaced by necrotic agents to represent fibers that were damaged during the mincing of fibers [35]. We assumed that the remaining 80% of the MMG fibers had normal function because published studies have reported a regenerative response in MMG treated injuries indicating that the muscle cells of the MMG are capable of normal behavior [13,82]. We assumed that the MMG fibers were parallel to the native muscle fibers for there was insufficient quantified literature data to make a more detailed assumption, and thus the MMG fiber cross sections were shown in the injury space. The model predictions were compared to the experimental results from the respective studies.

### Analysis of regeneration mechanisms

#### Pro-inflammatory & fibrosis perturbations

There has been a recent shift in the overall strategy for treating VML injuries, with recognition that modulating the early inflammatory response(s), as well as impairing nonproductive fibrotic pathways are also critical to improved tissue healing—as opposed to focusing solely on tissue engineering (i.e., replacing the lost tissue and cell populations) [8,21–23]. Thus, we utilized the ABM to predict the regenerative effects of modulating the pro-inflammatory and fibrotic cellular components of the tissue healing response, as well as altering the production of fibroblast-generated growth factors. We ran multiple “what-if” simulations to examine how each of the cellular behaviors in the ABM contributes to the tissue remodeling process. We individually varied the maximum number of pro-inflammatory macrophages, the maximum number of fibroblasts, and the secretion of fibroblast-secreted growth factors after VML injury by reducing them to 25%, 50% and 75% of baseline levels. In the model, the maximum number of pro-inflammatory macrophages and fibroblasts was reduced by preventing cells from proliferating or migrating into the simulation once the maximum number was reached. This kept the total cell counts below the limit for each perturbation. We tracked the fibroblast and SSC counts over time, the number of new fibers, and the amount of fibrosis as indicated by the model specific collagen density ratio to determine if these perturbations altered the ability of muscle fibers to regenerate following VML injury.

#### Combinatorial perturbations

After varying individual cellular behaviors, we then varied multiple parameters in a simulation to examine how the interplay between each of these behaviors leads to the tissue remodeling process. In model perturbations A and B, we limited the maximum number of M1 macrophages and fibroblasts by reducing to 75% of baseline levels, respectively. In model perturbation C, we eliminated the rule of microenvironmental cues for SSC proliferation and differentiation (see Table 2 for details) so that SSC proliferation and differentiation was no longer limited to being on a fiber edge and the ECM collagen levels no longer affected the SSC behavior. In model perturbation D, we lowered the threshold of the SSC differentiation signal. By lowering the threshold below zero, we were able to explore the possibility that the absence of pro-differentiation signals was contributing to the failed regeneration. We tested six different combinations of these model perturbations: (i) A + B + C + D; (ii) A + B + C; (iii) D; (iv) A + B + D; (v) A + C + D; and (vi) B + C + D. We tracked the fibroblast, SSC, and myotube counts over time, the number of new fibers, and the amount of fibrosis as indicated by collagen density to determine if the combination of perturbations altered the ability of muscle fibers to regenerate following VML injury.

## Results

### ABM simulated regeneration dynamics of VML injuries without repair

After tuning the unknown model parameters, listed in Table 4, the simulations predicted emergent cellular behaviors which were consistent with the findings of experimental studies of VML injuries in the absence of therapeutic repair. Fibroblast and myofibroblast fold changes peaked at day 7 and then plateaued from day 14 onward (Fig 4A) [9], consistent with experimental observations of col3a1 gene expression fold changes following VML injury [9]. SSC counts also peaked at day 7 and then remained elevated through day 28 (Fig 4B), which is also consistent with experimental observations in the literature [9]. The inflammatory cell dynamics captured the data available, including the sustained upregulation of many pathways associated with the inflammatory response [9,17,23] following VML injuries, as well as the previously reported overwhelming inflammatory M1 macrophage response compared to anti-inflammatory M2 macrophages (Fig 4C) [17,23]. Muscle fiber and ECM changes, including the reduced thickness of the muscle cross-section near and within the injury site, were also consistent with published experimental studies [12,68]. The ABM model for the baseline case of VML without repair predicted a complete failure in fiber regeneration and abundant collagen deposition in the injury defect (Fig 2) [11,17,18,35,36].

### ABM validated by comparing simulations to independent experimental results of 3 different VML treatments

#### Treatment 1

**Fig 5:**
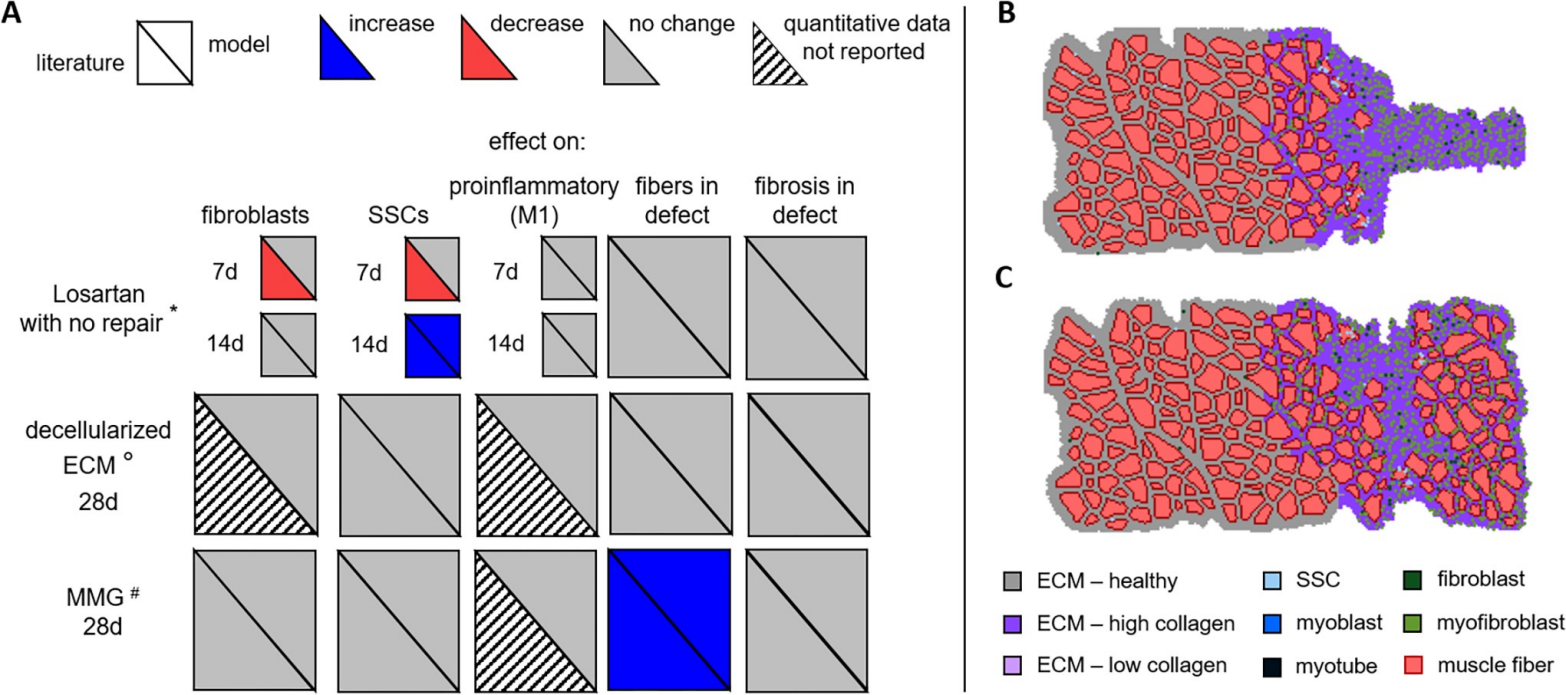
ABM predictions of cell behaviors, muscle fiber, and ECM changes 7, 14, and 28 days after injury and treatment were compared with published experimental results. Triangles represent an increase (blue), decrease (red), no change (grey), in response to the indicated treatment (i.e. Losartan with no repair, decellularized ECM, or MMG) or no quantified data available (striped) compared to VML injuries without repair (A). We compared quantitative changes in fibroblast, SSC, and pro-inflammatory macrophage numbers and compared qualitative changes in fibers and fibrosis in the VML defect. We also simulated VML injury treatments published in the literature and compared our model predictions to independent experimental results published in the literature: *[23], ° [11,13], ^#^[9,13,35]. Graphical ABM outputs at 28 days showed that treating the VML injury with decellularized ECM resulted in fibrotic tissue (i.e. increased collagen) filling the defect (B) and minced muscle graft (MMG) treatment resulted in muscle fibers present in the defect (C).

First, ABM simulations were run for the tissue response to VML repair following administration of Losartan. Losartan is an anti-fibrotic therapy that inhibits angiotensin II type 1 receptor activation and thereby blocks TGF-β1 [23]. Because our model does not specifically incorporate angiotensin II receptor blockade, we simulated the downstream effects of Losartan by reducing TGF-β production by myofibroblasts and macrophages 60% from baseline levels [79]. In this scenario, the ABM did not predict either the reduction in collagen III or the reduction in TGF- β fibrotic markers that has been observed experimentally following Losartan treatment 7 days after injury (Fig 5) [23]. However, consistent with experimental observations, the ABM predicted that Losartan treatment was associated with an increase in SSCs 14 days post-administration and injury. The ABM also predicted that by day 28 post injury, Losartan treatment causes collagen accumulation in the defect and no fiber regeneration, as has been reported in the literature [23].

#### Treatment 2

Next, we evaluated the ability of the ABM simulations to capture essential aspects of VML tissue repair following implantation of decellularized ECM. ABM simulations of decellularized ECM treatment following VML injury predicted an absence of new muscle fibers in the defect, indicating a lack of regeneration (Fig 5B), consistent with previously published experimental studies [11–13]. Further, the ABM predicted fibroblast, SSC, and M1 macrophage counts at day 28 post injury that were similar to those in VML injury simulations without repair (Fig 5A), and by day 28 the simulated defects contained increased collagen similar to VML injury simulations without repair (Fig 5B).

#### Treatment 3

Finally, model simulations of MMG treatment/implantation predicted similar fibroblast and SSC counts, accompanied by similar expression of fibrotic pathways, compared to VML injuries without repair at day 28 post injury, which is consistent with published experimental studies (Fig 5A) [9,13,35]. The ABM simulations of MMG treatment were also consistent with experimental observations in that both showed an increased number of muscle fibers by day 28 (Fig 5C) [9,13,35]. The model suggests that the new muscle fibers within the VML defect of MMG treated injuries resulted from the implanted muscle fibers within the MMG and were not generated by proliferation and differentiation of SSCs residing within the injured muscle [35]. The ability of our model to accurately predict repair using different treatments provides confidence in the utility of our model to capture tissue regeneration within VML injuries and points to some of the putative mechanisms underpinning experimental observations.

### ABM perturbations predict outcomes of potential VML treatments

#### Pro-inflammatory & fibrosis perturbations

**Fig 6:**
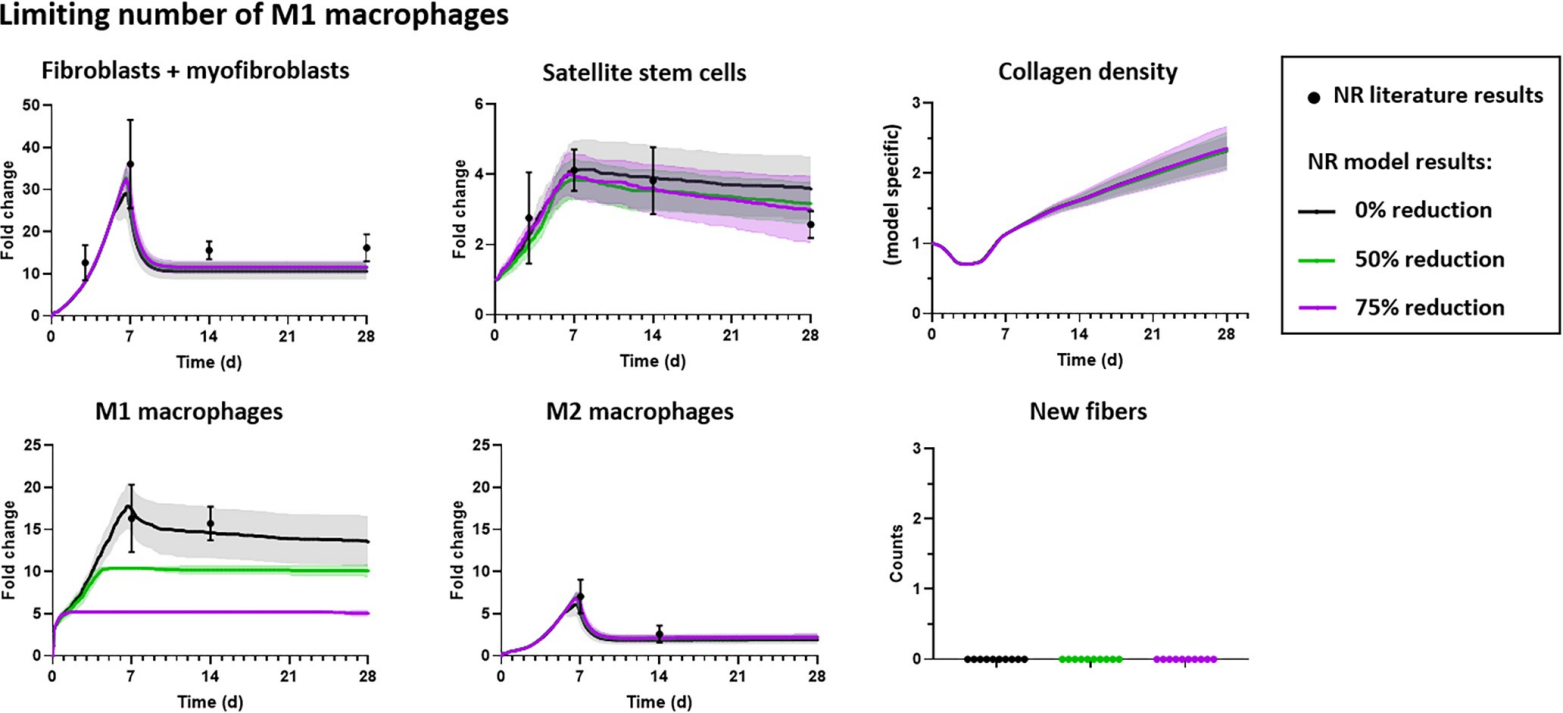
Limiting the number of M1 macrophages in the no repair (NR) ABM did not alter the amount of fibrosis as indicated by collagen density nor result in new muscle fibers filling the defect. The number of M1 macrophages was reduced to 50% and 75% of baseline levels. Outputs included fibroblast and myofibroblast fold changes, SSC fold changes, collagen density, macrophages fold changes, and number of new, regenerated fibers. Model results were reported as mean ± 95% confidence interval. Aguilar et al. 2018 experimental data of fibroblasts and SSCs reported as median ± standard deviation, and Garg et al. 2014 experimental data of M1 and M2 macrophages reported as mean ± standard error mean [9,23].

**Fig 7:**
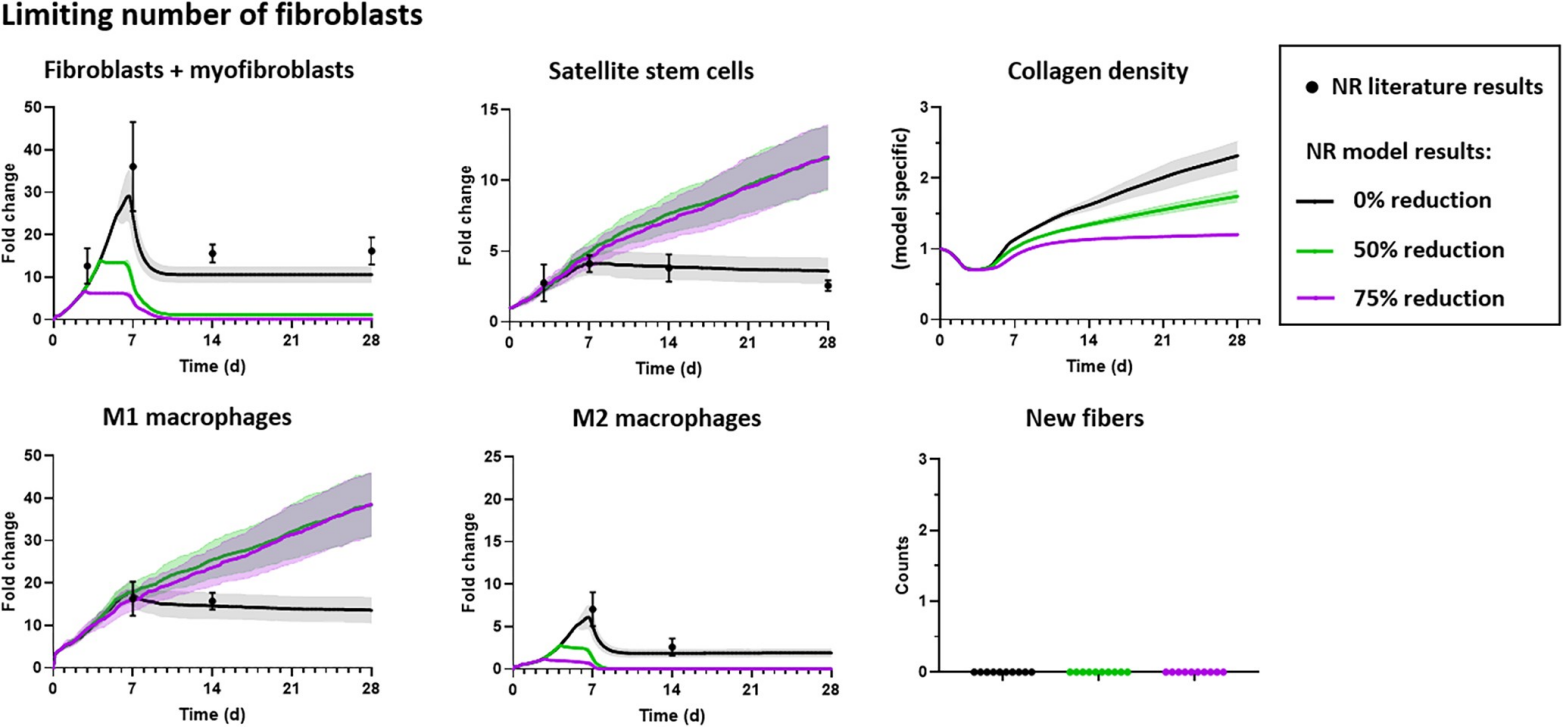
Limiting the number of fibroblasts in the no repair (NR) ABM reduced the number of fibroblasts and myofibroblasts following VML injury and altered the amount of fibrosis as indicated by collagen density. The number of fibroblasts was reduced to 50% and 75% of baseline levels. Outputs included fibroblast and myofibroblast fold changes, SSC fold changes, collagen density, macrophages fold changes, and numbers of new, regenerated fibers. Reducing the number of fibroblasts to 50% or 75% of baseline increased the number of SSCs and impaired the rate and extent of collagen accumulation in the defect. None of the perturbations to fibroblasts resulted in new muscle fibers filling the defect. Model results were reported as mean ± 95% confidence interval. Aguilar et al. 2018 experimental data of fibroblasts and SSCs reported as median ± standard deviation, and Garg et al. 2014 experimental data of M1 and M2 macrophages reported as mean ± standard error mean [9,23].

Simulations reducing the number of pro-inflammatory macrophages did not affect cellular behavior nor the amount of fibrotic tissue relative to baseline simulations (Fig 6). The cell curves in Fig 6 revealed that the other cell types included in the model were not compensating for the reduction in M1 macrophages and their curves were consistent with the baseline model. For example, while a 75% reduction in the number of M1 macrophages reduced their numbers to a level similar to M2 macrophages, that perturbation alone was not enough to alter the SSC differentiation signals and promote muscle regeneration. However, reducing the number of fibroblasts near the injury resulted in more SSCs and M1 macrophages (Fig 7). This anti-fibrotic perturbation increased the SSC proliferation signals resulting in larger fold changes of SSCs compared to the baseline no repair model. The reduction in fibroblasts translated to fewer cells depositing collagen which also led to less collagen accumulation.

**Fig 8:**
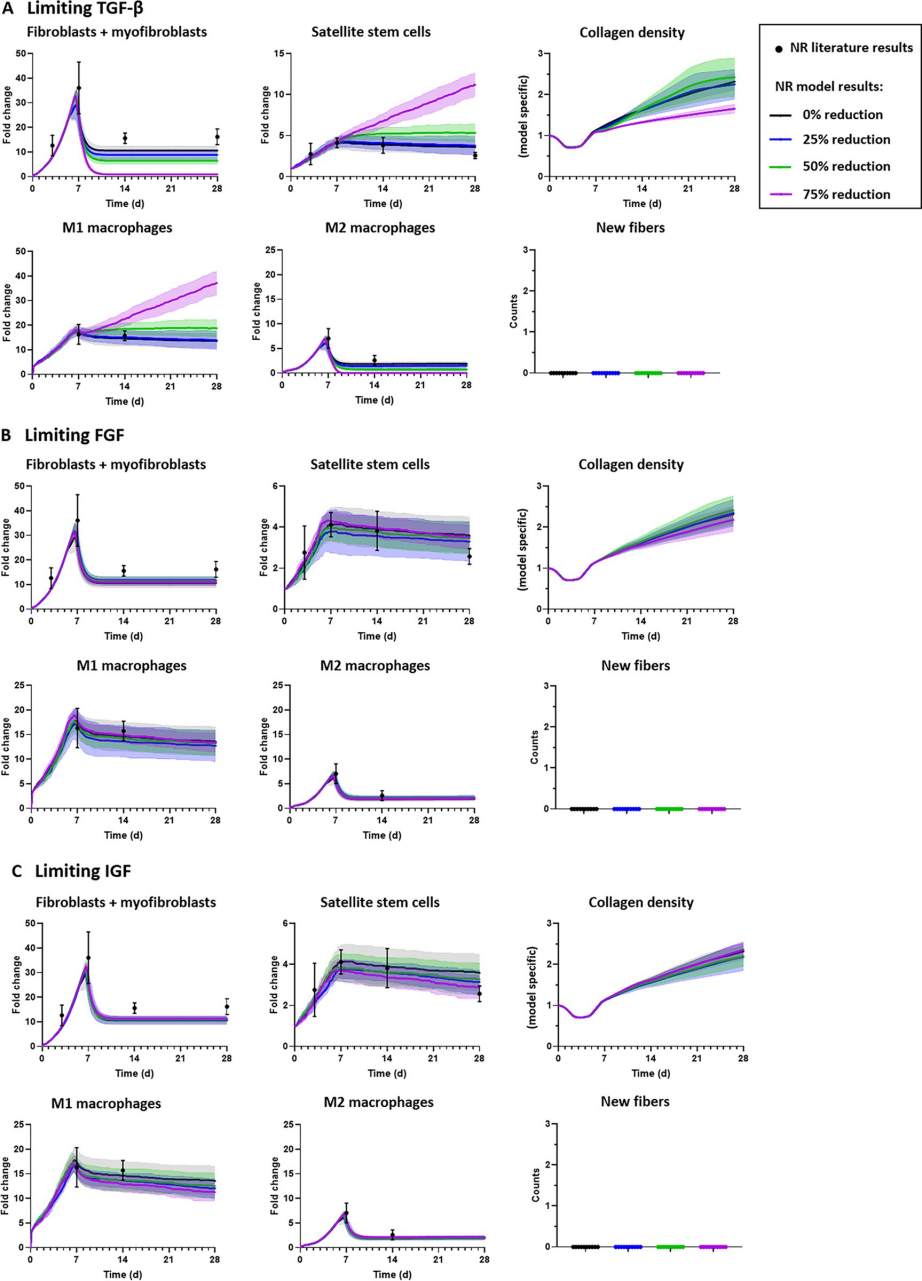
Limiting the amount of growth factors produced by fibroblasts in the no repair (NR) ABM revealed that only a large reduction in TGF-β levels altered the collagen density following VML injury. The levels of TGF-β (A), FGF (B), and IGF (C) were reduced to 25%, 50% and 75% below baseline levels, and fibroblast, SSC, and macrophage fold changes, collagen density, and counts of new, regenerated fibers were predicted. A 75% reduction of TGF-β secretion by fibroblasts increased the number of SSCs present and decreased the rate and amount of collagen accumulation (A). None of the perturbations resulted in new fibers filling the defect. Model results were reported as mean ± 95% confidence interval. Aguilar et al. 2018 experimental data of fibroblasts and SSCs reported as median ± standard deviation, and Garg et al. 2014 experimental data of M1 and M2 macrophages reported as mean ± standard error mean [9,23].

We simulated a reduction in the amount of fibroblast-generated growth factors (transforming growth factor beta, TGF-β; fibroblast growth factor, FGF; insulin-like growth factor, IGF) and tracked the cellular counts and collagen density over 28 days (Fig 8). A 75% reduction of TGF-β compared to baseline levels resulted in higher SSC counts and lower collagen density (Fig 8A). In comparing the 75% reduction of TGF- β simulation to the Losartan simulation, both simulations resulted in higher SSC counts after 10 days post-injury; however, the 75% reduction of TGF- β resulted in zero fibroblasts and myofibroblasts by 14 days post-injury but the Losartan simulation continued to have similar fibroblast and myofibroblast cell counts as the no repair baseline simulation (Figs 5A and 8A). This suggests that impairing fibroblast recruitment, and/or severely inhibiting TGF-β production following injury, may improve the ability of the muscle to repair following VML injury since there are more SSCs present and less fibrotic tissue, marked by lower collagen density, in the injury. Nonetheless, the ABM predicted no new fibers in the defect as a result of reducing TGF-β production nor reducing the other fibroblast-generated growth factors or cell populations (Figs 6–8), thus suggesting that in isolation this treatment approach would be insufficient to regenerate the muscle lost to VML injury.

#### Combinatorial perturbations

**Fig 9:**
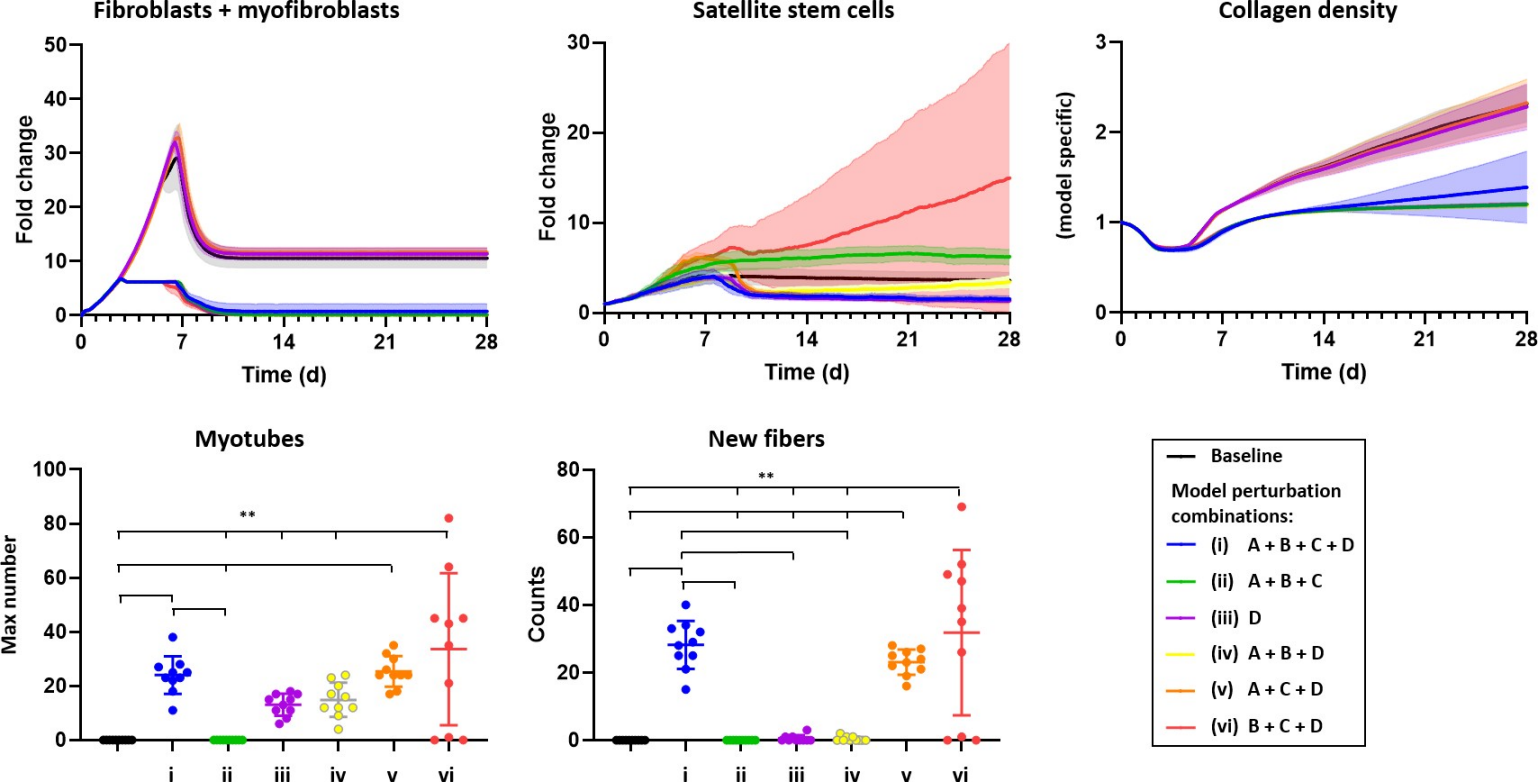
A combination of model parameters, reflecting the key biological aspects of failed regeneration in VML injuries, were adjusted in combination to determine how these perturbations affected collagen density and new muscle fiber infiltration into the defect. In perturbation “A”, the maximum number of M1 macrophages and in perturbation “B”, the maximum number of fibroblasts were reduced to 75% of baseline levels. In perturbation “C”, SSC migration behaviors were adjusted such that SSCs preferred being in isolation on ECM as opposed to in their niche next to a fiber. Model perturbation D, which corresponded to the threshold for SSC differentiation into myofiber, was reduced so that SSC differentiation was more frequent. When perturbations, C and D, were implemented simultaneously, then there was a significant increase in the number of myotubes and new fibers. ** p < 0.01, statistical significance between groups using a one-way analysis of variance and Holm-Sidak post hoc test.

We then simulated VML treatments that incorporated a combination of different approaches (Fig 9). The fibroblast count and collagen density curves corresponded directly to whether model perturbation B (limiting the number of fibroblasts) was implemented. When the fibroblast counts were limited, less collagen was accumulated. For SSC counts, the simulations with model perturbation D (lowered SSC differentiation threshold) resulted in fewer SSCs because SSCs differentiated and no longer contributed to the number of SSCs. Combination vi simulation (perturbations B, C, D) had the largest number of SSCs and largest 95% confidence interval. Model perturbation B (decreased fibroblast counts) led to larger SSC proliferation signals resulting in more SSCs, which is consistent with observations of Fig 7. However, when model perturbation A (limiting M1 macrophages) and B were implemented simultaneously, the SSC proliferation signal did not increase exponentially and the SSC counts plateaued. For markers of muscle regeneration, increased myotube (fully differentiated SSCs) counts correlated with the presence of model perturbation D (lowered SSC differentiation threshold). Interestingly, the largest myotube counts and number of new fibers occurred with the combination of model perturbation C (altering SSC microenvironmental cues) and D (lowered SSC differentiation threshold). In these simulations (combinations i, iv, vi), the SSCs are in a better location and environment to generate new fibers and there was improved regeneration as marked by a significant increase in the number of new fibers (Fig 9).

**Fig 10:**
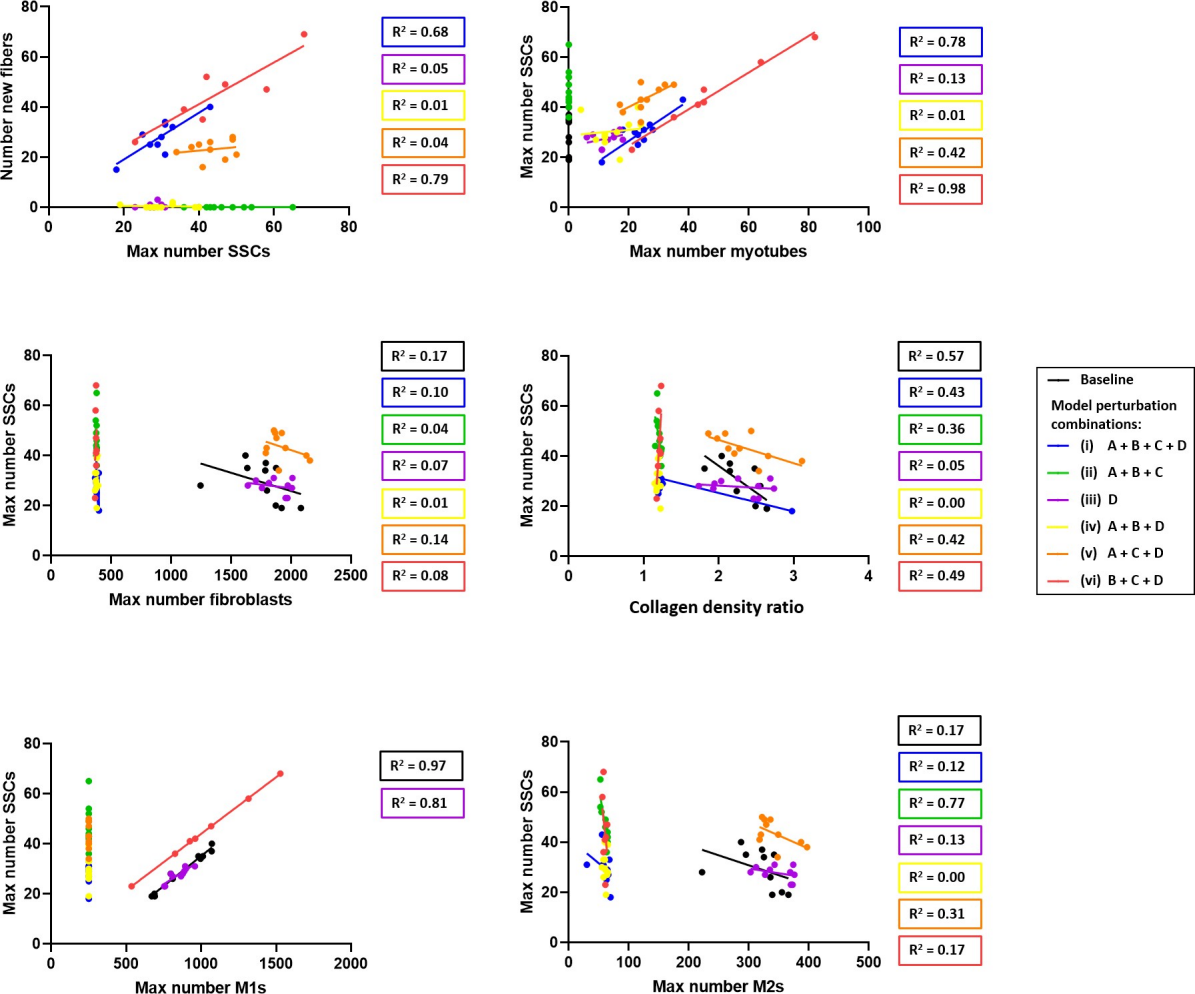
In model perturbation combinations i and vi, the number of new fibers correlated with the maximum number of SSCs. In model combinations i, v, and vi, the maximum number of SSCs correlated with the maximum number of myotubes. In perturbation “A”, the maximum number of M1 macrophages and in perturbation “B”, the maximum number of fibroblasts were reduced to 75% of baseline levels. In perturbation “C”, SSC migration behaviors were adjusted such that SSCs preferred being in isolation on ECM as opposed to in their niche next to a fiber. Model perturbation D, which corresponded to the threshold for SSC differentiation into myofiber, was reduced so that SSC differentiation was more frequent. R^2^ values quantify the goodness of fit for linear regression. If R^2^ values are not shown, then the linear regression was a perfect line (R^2^ = 1). ROUT analysis was used to identify outliers for each model combination and three outliers were identified in combination vi.

We further analyzed the relationship between predictions from the model perturbation combinations to investigate which biological aspects were driving the perturbation results. First, we focused specifically on the correlation of new fibers for they were a marker of improved muscle regeneration. The number of new fibers correlated with the maximum number of SSCs in model perturbation combinations i and vi (Fig 10). These perturbation combinations were the only combinations which incorporated perturbations B (decreased fibroblast counts), C (altering SSC microenvironmental cues), and D (lowered SSC differentiation threshold). The initialization was not different in any of the model combination simulations; thus, the predicted increase in number of new fibers were emergent model predictions that resulted from the model perturbations. The maximum number of SSCs correlated with maximum counts of myotubes in model perturbation combinations i, v, and vi (Fig 10). These perturbation combinations were the combinations which incorporated both SSC behavior perturbations (C–altering SSC microenvironmental cues, and D–lowered SSC differentiation threshold). In model combinations without limiting the number of M1 macrophages, there was a strong correlation between peak SSCs counts and peak M1 macrophages counts.

## Discussion

The goal of this study was to develop a computational model that predicts muscle regeneration and tissue remodeling following VML injury and/or treatment. By incorporating literature-derived rules from over 100 sources, the ABM was able to capture the autonomous behaviors of fibroblasts and SSCs and simulate regenerative dynamics that were not explicitly defined in the model. We simulated the regenerative response of unrepaired and treated VML injuries, and then we used the model to explore the outcomes of potential therapies and probe mechanisms underlying regeneration following VML injury. One of the fundamental findings of our study is that the model simulations suggested that multiple aspects of SSC behaviors are responsible for the overt failure of tissue repair in the setting of an untreated VML injuries. Moreover, consistent with existing preclinical and clinical data, the model simulations also indicate that vastly improved muscle regeneration and thus functional outcomes for VML injury cannot be affected by treatments that address individual aspects of the tissue healing response (Figs 6 and 7).

To this end, we conducted theoretical simulations (combinatorial perturbations, Fig 9) which predicted that simultaneous alterations in multiple cellular behaviors produced significantly improved muscle regeneration in the VML defect. We explored the impact of the following on muscle regeneration: reducing the presence of pro-inflammatory cells (perturbation A) and fibroblasts by 75% (perturbation B) and altering the SSC microenvironmental cues required for SSC differentiation (i.e., location on a fiber edge, or on ECM with native healthy stiffness–model perturbation C), and addressing the absence of pro-differentiation SSC signals in the VML repair environment in model perturbation D. The model simulations suggested that the simultaneous combination of removing SSC dependency on its microenvironment for differentiation and lowering the threshold for SSC differentiation so that it occurs more frequently, will significantly increase the number of new fibers in VML defects (Fig 9). Additional model analysis indicated SSC behaviors contribute to failed SSC differentiation and repair of damaged muscle fibers on the one hand; while, SSC behaviors in combination with large fibroblast counts contribute to the failed regeneration of new muscle fibers in the VML defect on the other (Fig 10). Overall, the model indicated that SSC behaviors were the most important aspect of failed regeneration in VML injuries. Although it is not currently possible to implement all of these changes experimentally, the model allowed us to examine if, and how, addressing multiple cellular behaviors in combination would alter muscle regeneration in VML injuries.

It is important to address the simplifying assumptions and limitations of our model. In developing the ABM, our goal was to incorporate fiber regeneration and fibrosis, which are the most prevalent and investigated aspects of repair in VML injury, but we did not incorporate other aspects of muscle structure and function that are known to be affected during regeneration. For example, our ABM does not include neuromuscular junctional changes, microvascular network adaptations, the effect(s) on muscle function and activity (i.e., contraction), or different muscle fiber types. We have focused on a subset of cells (fibroblasts, satellite cells, and macrophages), but there are other cell types present in muscle (e.g. fibroadipocytes, pericyte cells, angioblasts, lymphocytes). Another simplifying aspect of our ABM is that inflammatory cells were incorporated as a system of ODEs. In the future, the ABM could be extended to incorporate inflammatory cell behaviors including interactions with other cell types. For cell types that were modeled, we incorporated relatively simple cellular behaviors and interactions through a series of probability defined rules; as opposed to modeling intracellular behaviors such as individual binding receptors, binding rates, etc. In the future, the ABM can be expanded to incorporate the biophysical environment of the cells and cell mechanics explicitly as well as coupling the ABM with signaling focused models to capture the details of molecular mechanisms in cell dynamics [83,84]. We have also assumed that the presence of growth factors is an important aspect of tissue regeneration following VML injury and have assumed evenly distributed growth factors, consistent with other ABMs [28,29,33]. We have not incorporated diffusion mechanics because there are currently no studies quantifying growth factor regional distributions in VML injuries. Future experimental studies quantifying growth factor diffusion would provide the data to incorporate diffusion mechanics in the ABM. Additionally, future studies that expand upon the current model could incorporate more complex behaviors of fibroblasts, different types of collagen, and behaviors of collagen thickening and scarring to more accurately capture and predict fibrotic changes after VML injury.

The development of the model is also limited by the availability of experimental data. The model was primarily informed by the few VML experimental studies that have focused on cell counts at time points less than 2 months post-injury as well as the many studies that have focused on the morphology of the tissue and functional response at longer time points of 3 and 6 months [11,12,16,18]. For the cell types that were modeled, we were limited to defining the cells by the markers and methods used in these experimental studies. The experimental data we used for comparison with the model outputs was quantified using gene expression from bulk tissue. A limitation of this method is that many cell populations have overlapping transcriptional profiles. To allow us to compare our model cell outputs to quantified experimental data, we assumed that cell markers were for specific cell populations. For example, we assumed that CCR7 was a marker for M1 macrophages, consistent with other studies [23], but CCR7 is also expressed by T cells and dendritic cells. Additional experimental studies quantifying a larger range of cell types would allow us to refine our model to further ensure that behaviors of specific cells of interest/study are not being compensated for by another cell type. Also, expanding our ABM to couple with a molecular signaling model would allow heterogenous cell populations to be incorporated.

We also had to make assumptions regarding the spatial distribution of cells because this has not been quantified in experimental studies. For example, we had access to experimental data quantifying the number of M1 and M2 macrophages following VML injury but there have been no efforts to describe the spatial concentrations of M1 and M2 macrophages over time [13]. Our ABM represents the higher proportion of M1 macrophages compared to M2 macrophages that has been observed experimentally, and we have assumed a spatial distribution of both M1 and M2 macrophages that places 60% in the border region and 40% in the healthy region [17]. Additional experimental studies are needed to confirm the model predictions about the spatial distribution of cellular behaviors. We have modeled one two-dimensional geometry of the muscle defect, but future work can expand the model to represent three-dimensions, which will allow this model to be applied to other VML animal injury models, such as injury to the rat tibialis anterior [19,20]. Additionally, the source of migrating cells into the defect space could be added to the model providing additional insight into the effect of injury geometry on the tissue response.

In the simulations of no repair and decellularized ECM treated VML injuries, the thickness of the muscle cross-section near, and within, the injury site decreases. This prediction is a result of an ECM agent rule that two neighboring ECM agents with low collagen have a probability of merging into a single agent and is consistent with experimental observations [67]. The simulations of VML injuries treated with Losartan did not recapitulate the early cellular dynamics that have been reported experimentally; however, the model was consistent with data collected at later time points that describe cellular changes and failed muscle regeneration in the VML defect [23]. The Garg et al. 2014 experimental study, for example, reported reduced deposition of collagen type I and no changes in collagen type III in Losartan treated injuries compared to VML injuries without repair [23]. We have incorporated a simplified representation of fibroblast and collagen behaviors, and we did not incorporate collagen subtypes into this ABM nor did we incorporate the specific mechanistic action of Losartan (see method section). This generalized behavior in our model likely explains why it failed to capture the early cell dynamics; however, in future work, the model can be expanded to represent mechanistic behaviors [84,85]. However, the model’s ability to predict cellular changes seen at later time points supports its utility in predicting longer-term remodeling outcomes (i.e. scarring and regeneration), which would be helpful for designing new therapeutic approaches.

With these limitations in mind, the demonstrated ability of the ABM to predict the effects of various treatments for VML injuries motivates its deployment in future studies of novel therapeutics. That is, the model can be utilized to predict the effectiveness of a new therapeutic and aid in the design of more effective therapies to limit the number of experiments that would otherwise need to be conducted. For example, a cell-seeded biomaterial could be simulated in the model and the material’s degradability and initial cell density could be optimized through model perturbations. The ABM can also be used to inform the design of experiments by identifying the most critical time points and outcomes to examine in an experimental study—again, perhaps saving the time and expense of unnecessary initial experiments. On a broader scale, computational modeling enables improvements in the design of therapeutics for VML injuries by guiding injury specific treatment options. Finite-element (FE) models can inform therapeutic design based on mechanistic insight of force transmission [12,86], and now ABMs can be used to inform therapeutic design using cellular mechanistic insight. Furthermore, hybrid models that couple FE modeling with ABMs offer a unique ability to explore the interactions between biomechanical and biochemical mechanisms of muscle regeneration [87,88]. In conclusion, this work demonstrates that an ABM of regeneration following VML injury provides important new insight into the cellular mechanisms governing wound healing and repair. Utilizing computational tools to inform tissue engineering and regenerative medicine therapeutics has the potential to drive more rapid and efficient clinical translation of regenerative therapeutics for VML injuries.

## Supporting information

S1 Fig Updated model preventing cell-cell overlap does not significantly influence the model outputs. The updated model checks that there are no other cellular agents on a new location before moving a cellular agent. The model code available on SimTK includes prevention of cell-cell overlap.
(TIF)

S1 Video Animation video of ABM of VML injury regeneration without repair. (MP4)

S1 Table Inflammatory cell ODE parameter iterations after tuning using the genetic algorithm (GA) and manual adjustment. The GA objective function was the sum of squared differences between simulation results (subscript ABM) and experimental data (subscript EXP) (Eq 3).
(TIF)
