# Supplementary figures and images for: Agent-based model provides insight into the mechanisms behind failed regeneration following volumetric muscle loss injury
Source: PLoS Comput Biol. 2021 May 10;17(5):e1008937. doi: 10.1371/journal.pcbi.1008937 (PMC8110270; doi:10.1371/journal.pcbi.1008937)

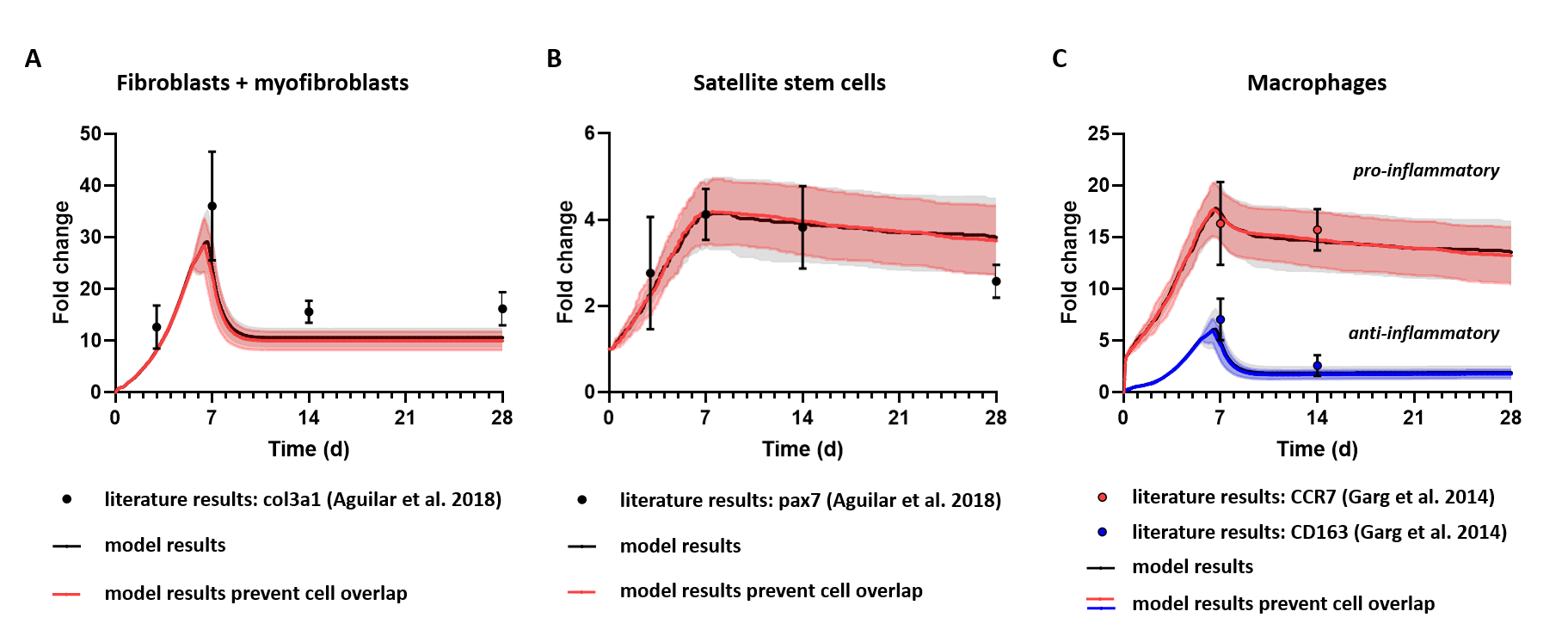

Supplement: S1 Fig — The updated model checks that there are no other cellular agents on a new location before moving a cellular agent. The model code available on SimTK includes prevention of cell-cell overlap. (TIF) [file pcbi.1008937.s001.tif]

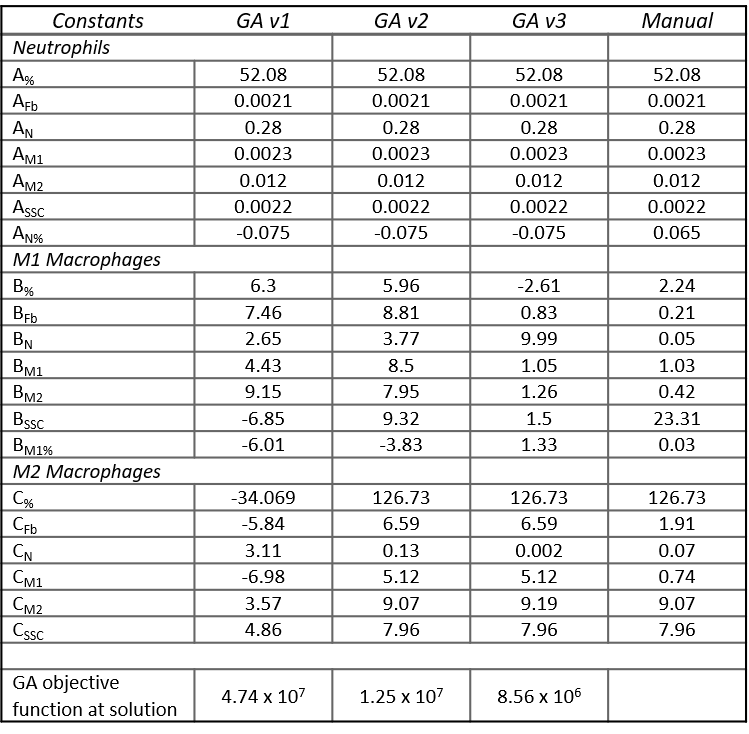

Supplement: S1 Table — The GA objective function was the sum of squared differences between simulation results (subscript ABM) and experimental data (subscript EXP) (Eq 3). (TIF) [file pcbi.1008937.s003.tif]
